# A Structural Barrier to Healthcare: Stigma of Alcohol and Substance Use Disorders Among Health Care Workers

**DOI:** 10.1192/j.eurpsy.2022.2140

**Published:** 2022-09-01

**Authors:** İ.G. Yilmaz-Karaman, T. Gündüz, G. Güleç

**Affiliations:** 1 Eskişehir Osmangazi University, Faculty of Medicine, Psychiatry Department, Eskişehir, Turkey; 2 Eskişehir Osmangazi University, Faculty of Medicine, Psychiatry, Eskişehir, Turkey

**Keywords:** substance use disorder, Alcohol use disorder, stigma, Addiction

## Abstract

**Introduction:**

Individuals with substance use disorders are considered unpredictable and violent by the public. Besides, health care workers (HCW) may have negative attitudes towards them, despite their knowledge about addiction; which is related to lower quality of care. In Turkey, addiction service users are predominantly male, over ninety percent; while women make up a large percentage of psychiatrists.

**Objectives:**

The present study aims to evaluate if the HCWs level of stigma towards individuals with substance use disorder changes due to gender and mental health sector experience of the HCWs.

**Methods:**

Within an online survey, participant HCWs answered Attitudes Towards Treatment of Substance Use Disorders Scale, Substance Addiction Stigmatization Scale, Alcohol Addiction Stigmatization Scale; in addition to sociodemographic questions.

**Results:**

Three hundred ninety-eight HCWs were included in the analyses. 22.7% of them (n=91) were recruited in mental health sector. Mental health care workers had lower levels of stigma towards individuals with alcohol use disorders (14.78 vs 16.21, p=0.048) and substance use disorders (14.21 vs 20.09, p<0.001) and, lower levels of stigma towards addiction treatments (20.89 vs 23.93, p=0.007). Among mental health care workers, women scored higher numbers of stigmatization towards alcohol use disorder and addiction treatments (16.26 vs 12.98, p=0.003; 23.84 vs 17.29, p<0.001). On the other hand, women and men in other HCWs groups did not differ from each other in terms of stigmatization measurements (See Figure 1)

**Figure:**
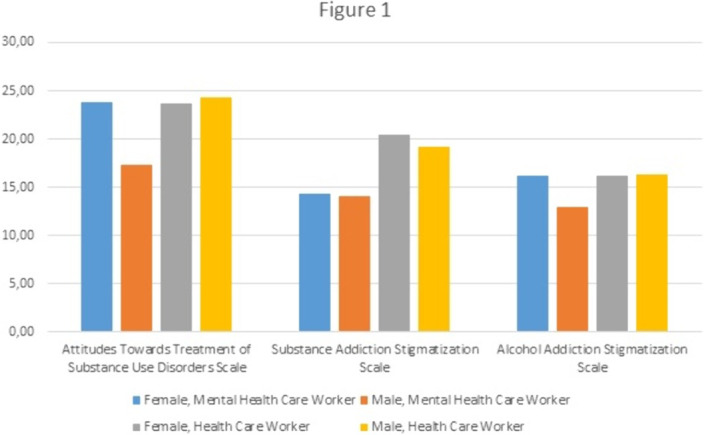

**Conclusions:**

The gender of mental health care workers may be related to stigmatization towards addictive disorders. Future research should evaluate underlying factors.

**Disclosure:**

No significant relationships.

